# Supplementary material for: The presence of spin in systematic reviews focused on diabetic neuropathy: A cross-sectional analysis
Source: PLoS One. 2022 Sep 26;17(9):e0274744. doi: 10.1371/journal.pone.0274744 (PMC9512415; doi:10.1371/journal.pone.0274744)
Supplement: Attachment — Submitted filename: Response to Reviewers.docx [file pone.0274744.s001.docx]

Response to reviewers

Reviewer #1: Khan and colleagues performed a systematic review to evaluate the presence of nine of the most severe types of spin (as identified by Yachitz et al.) in the abstracts of systematic reviews on diabetic neuropathy. This work builds upon a number of similar evaluations of spin of a variety of disciplines by their group, and therefore the results can be compared to other research topics, which they have done to an extent in the discussion. The methods appear to be rigorously executed from what is provided, although not all materials are able to be evaluated by the reviewer to make a final determination (see first comments).

Major comments

1."All relevant data are within the manuscript and/or Supporting Information files"

- Unless I missed it, I do not see raw data available to review.

We will attach raw data to our revised submission in order for the data to be reviewed. We shall also make the protocol available to the public.

Line 86: "To ensure our study’s reproducibility and transparency, this study’s protocol, extraction forms, and data analysis scripts can be found in Open Science Framework (OSF) [link]."

- This link is not active (permission error) for the reviewer to check these items. Please allow public access or attach the items for review if there is a reason for these items not to be public before publication.

We have made the protocol available to the public.

PRISMA flow diagram

- Please provide enough information so a reader can reproduce your process of using automation tools that were used to screen records, and so readers can assess the reliability of doing this.

The PRISMA flow diagram is updated to where the reader will be able to reproduce the process of screening records.

Do the following statements contradict one another?

- "Since the final sample size of 114 was not sufficiently powered to do multivariable logistic regression, we calculated unadjusted odds ratios which evaluated relationships between spin presence within abstracts and study characteristics."

- "Our logistic regression model found no significant association between abstracts with spin and a study’s PRISMA adherence, intervention type, if the publishing journal recommends PRISMA adherence, funding source, or the 5-year impact factor of the publishing journal (Table 2)."

We edited the logistic regression model statement by saying there was a significant association of the presence of spin and study characteristics based on the 95% CI of the odds ratio being either above or below.

Minor comments

Abstract

- Conclusion: I agree that compared to other fields, review abstracts on diabetic neuropathy seem to contain less spin. Yet, 8% of studies still seems unacceptable, and therefore I would not use the language that they "accurately portray the findings of the review's full-text". Your conclusion (line 211) seems to agree that this is encouraging but we should not be complacent.

We agree with the reviewers comment and changed this statement in the conclusion to “When comparing our results to other fields of medicine, systematic reviews related to diabetic neuropathy more often accurately portray the findings of the articles’ full-text. Given that nearly 8% of these systematic reviews did contain spin, further reducing spin would lessen potential bias within these articles, which may be beneficial to other researchers, healthcare providers, and their patients.

Line 69: "physicians must stay updated through research and various learning platforms".

- Do you mean by reading scientific literature?

We changed various learning platforms to “scientific literature”.

70: "Comprehensive study analyses which comprise multiple studies" -> change to "Systematic reviews and meta-analyses", or rephrase another way.

We directly changed this statement to “Systematic reviews and meta-analyses serve as an excellent method for physicians to keep their medical knowledge current”.

Reviewer #2: Thank you for the opportunity to review the manuscript entitled “The Presence of Spin in Systematic Reviews Focused on Diabetic Neuropathy: A Cross-Sectional Analysis”.

The authors have performed a meta-research study that consisted of a systematic search for systematic reviews on diabetic neuropathy treatment and an analysis of the prevalence of different types of spin. They also extracted review characteristics and assessed the included review’s methodological quality using AMSTAR 2, and then evaluated whether there was an association between these characteristics or quality and the prevalence of spin.

While this is an important field of study and valuable research question, there are several aspects concerning the manuscript that should be addressed:

Major:

1. Apart from AK, HR, DW and MV, it is currently unclear how the co-authors were involved in the study process. Please provide an author statement using the CRediT Taxonomy.

We have used CRediT Taxonomy to provide an author statement.

2. Introduction: Argumentation:

a. I cannot entirely follow the argumentation around systematic reviews, e.g. “Because systematic reviews are the gold standard it is important that they are conducted without bias or misleading findings”. I would consider it the other way around: By definition, systematic reviews use explicit, systematic methods to reduce the risk of bias in the review process (bias cannot be eliminated), therefore they are considered the highest level of evidence (for most questions). Given this high standing, they are supposed to report findings in a way that is not misleading. However, while spin has long been known to occur in RCTs, there is now evidence for spin in systematic reviews, too (citations of such studies).

We changed the argumentation of systematic reviews directly to the reviewer’s suggestion to strengthen our argumentation around systematic reviews. The paragraph now states “Systematic reviews use explicit, systematic methods to reduce the risk of bias in the review process, therefore they are considered the highest level of evidence. Given this high standing, they are supposed to report findings free of spin.

b. The concept of spin should be elaborated more in the argumentation, e.g. introduce the classification by Yachitz before the aims & objective statement.

We elaborated the concept of spin earlier in our introduction. We stated, “Yachitz et al. classify spin based on three overarching categories: misleading reporting, misleading interpretation, and inappropriate extrapolation.” [6]. We also altered our paragraph to state examples of spin throughout the introduction in order to illustrate the use of spin in various fields. We stated, “Spin has been identified as a broadly used form of bias in randomized controlled trials within various fields, as well as, specific disease states. [[8,](https://paperpile.com/c/I3oh8f/w133y+Xepnj) 9] However, discrepancies have been found between specialties. For example, 70% of otolaryngology abstracts contain spin in comparison to 44.3% of emergency Medicine abstracts, and 27.3% of cardiology abstracts. Due to the variability in results of past research, further exploration is warranted to examine the presence of spin in additional fields”. We also attempted to introduce the classification by Yachitz et. al in the abstract.

c. The point about physicians often only reading abstracts should also be moved up to the argumentation, i.e. before the aims & objective statement.

We attempted to state our point of physicians often only reading abstracts earlier in the introduction. We stated, “While the entirety of a scientific work should maintain a high professional standard, there is one section that should be more thoroughly scrutinized - the abstract. As the portion most commonly read by clinicians, the abstract has the greatest potential to alter the course of patient treatment and outcomes.” [7]

3. Introduction: Choice of references: It is unclear why spin in anesthesiology and emergency medicine RCTs is mentioned here; I would suggest citing studies of spin in RCTs on diabetic neuropathy treatment, if available, or an evaluation of spin in RCTs that is not focused on a specific field. If there is a reason to believe that the medical field influences the amount of spin, it would be interesting to cite such evidence in the introduction. Regarding spin in abstracts of systematic reviews: it would be interesting to present a brief overview of the findings of the other meta-research studies conducted by the team around MV (at least 18 of which have already been published according to a PubMed search).

Our goal with referencing different studies was to elaborate how variable the presence of spin is for various fields of medicine. To our knowledge there are not any available studies illustrating the use of spin in RCTS on diabetic neuropathy. This was the goal of our study. The additional goal of referencing different fields of medicine was to show how there has yet to be a study examining the presence of spin in the field of diabetic neuropathy. We altered our paragraph to say, “Spin has been identified as a broadly used form of bias in randomized controlled trials within various fields, as well as, specific disease states. [[8, 9](https://paperpile.com/c/I3oh8f/w133y+Xepnj)] However, discrepancies have been found between specialties. For example, 70% of otolaryngology abstracts contain spin in comparison to 44.3% of emergency Medicine abstracts, and 27.3% of cardiology abstracts. Due to the variability in results of past research, further exploration is warranted to examine the presence of spin in additional fields. One unexplored topic is diabetic neuropathy and the presence of spin within treatment options. As diabetic neuropathy is highly prevalent in the US, the evaluation and maintenance of scientific integrity are vital.”

4. Methods: Protocol and Reporting: The files in OSF do not appear to be public (when I tried it (as a logged-in OSF user), it said “You Need Permission”). Please make your files public, otherwise the reproducibility and transparency of your study (which you name as a strength) is not given.

We have now made the files public. We have also now placed a hyperlink in order to access the protocol quicker.

5. Methods: Protocol and Reporting: Please provide more information on the third-party analytical team.

This would be our additional statistical analysts. They verified the statistical scripts and reporting of results.

6. Methods: Protocol and Reporting: While I greatly encourage the use of reporting guidelines, it is unclear to me why PRISMA 2020 was used for this meta-research study. It also appears to me that there were some misunderstandings, i.e., in the PRISMA checklist it says “Yes/Table 3” for the items 13e (Describe any methods used to explore possible causes of heterogeneity among study results (e.g. subgroup analysis, meta-regression)) and 14, neither of which was applicable in this study. Furthermore, line numbers need to be updated if use of PRISMA 2020 is continued (or changed to page numbers may be more feasible).

We initially provided a PRISMA guideline in order to increase the transparency of our study. After much discussion we have elected to remove the PRISMA 2020 considering the type of study we were conducting.

7. Methods: Data Extraction: The column in Table 1 that contains results must be reported in the results section please. The remaining table would benefit from examples and should contain the citation of Yachitz et al.

We changed the placement of table 1 to the results section. We also included the citation for Yachitz et al. After much discussion we thought it would best serve the manuscript if our examples remained in the discussion portion.

8. Methods: Data Extraction: The description of AMSTAR-2 assessment should be an extra paragraph containing an explanation of how the overall assessment was derived at. Furthermore, the tool needs to be cited correctly (https://www.bmj.com/content/358/bmj.j4008) and it is currently unclear what you mean by “high construct validity coefficients based on the original AMSTAR-2 instrument (r = 0.91) and the (r = 0.84).[16]”.

We changed our citation to https://www.bmj.com/content/358/bmj.j4008. We removed the previous statement of “high construct validity coefficients based on the original AMSTAR-2 instrument (r = 0.91) and the (r = 0.84)” to “the AMSTAR-2 has high reliability and validity compared to other systematic review appraisal tools, such as the original AMSTAR and Sach’s Instrument.”[18]

9. Methods: Data Extraction: Please provide the categorization system for each characteristic and add that you also extracted the date the journals received the systematic reviews.

We attempted to clarify the study’s intervention type and the funding source. We also added the date the journals received the systematic reviews.

10. Results: Characteristics: Please provide a list of included reviews as well as a list of exclusions with reasons.

We updated the PRISMA flow diagram to include more information of the inclusion and exclusion criteria, and we will be providing a list of all included and excluded reviews.

11. Results: AMSTAR-2 Rating: Please provide the results per individual review in a supplemental file.

We will be providing our raw data sheets from AMSTAR for transparency.

12. Discussion: Please discuss potential reasons why systematic reviews focused on the management of diabetic neuropathy tend to have less spin than systematic reviews from other fields.

We are not sure why systematic reviews focused on the management of diabetic neuropathy tend to have less spin than systematic reviews from other fields. Our study was designed to be purely objective. Additionally, our results show no relationship between study characteristics and the presence of spin.

13. Discussion: It is unclear why Ottwell et al. and Heigle et al. are being cited. It would be more informative to report the range of spin across the systematic review abstracts of various clinical disciplines that the team around MV have investigated.

We removed the citation and elected to place the presence of spin across the systematic review abstracts of various clinical disciplines within the introduction.

Minor:

14. To better differentiate between studies as in other meta-research studies and the included articles, I would suggest to re-word them to “included reviews”, “analyzed reviews”, etc.

We re-worded the included articles to ‘included reviews’ in order to separate the other meta-research studies from what articles we included in the study.

15. Abstract: Methods: “…, of which included 114 systematic reviews for spin assessment.” Suggest to insert “we” in front of “included”.

We applied this edit, so it now reads as “of which we included 114 systematic reviews for spin assessment.”

16. Abstract: Methods: Please state that you mean the nine most severe spin forms as defined by Yachitz et al. 2016.

We cited Yachitz et al. 2016 in the abstract.

17. Abstract: Methods: Please include a brief description of the analyses performed.

We clarified the analyses performed by stating “Since the final sample size of 114 was not sufficiently powered to do multivariable logistic regression, we calculated unadjusted odds ratios which evaluated relationships between spin presence within abstracts and study characteristics.”

18. Abstract: Results: “No association exists”, please reword to “was observed”.

We reworded “no association exists” to “was observed”.

19. Abstract: Conclusions: Please change to “whose abstracts mostly portray the findings of the review’s full-text accurately.” to reflect the results adequately.

We changed the abstract's conclusion by adding “When comparing our results to other fields of medicine, the field of diabetic neuropathy research publishes systematic reviews whose abstracts mostly portray the findings of the review’s full-text accurately to reflect the results adequately”.

20. Methods: Search Strategy: Please rename the section to “search strategy and screening” and describe if there were any limits to the search and how you used the automation function of Rayyan.

We renamed Search Strategy to “Search strategy and screening,” and we explained “One of us (DW), a systematic review librarian, systematically searched Embase and MEDLINE to locate systematic reviews limited to the management of diabetic neuropathy treatment (Fig 1).

21. Methods: Fig 2 is mentioned before Fig 1, please correct.

We corrected the figure placement.

22. Results: Figure 1: It should “diagram” not “diaphragm”. Please provide a legend for the asterisks and correct the number of records identified from databases (= MEDLINE and Embase) to 1297 and the number of records identified from registers to 0.

We corrected the spelling of figure 1 and provided a legend for the figure.

23. Results: Characteristics: “The primary type of intervention was pharmacological therapies (77/114, 67.5%), followed by non-pharmacological interventions (37/114, 32.5%), and nonsurgical interventions (0/114, 0%).” Do you mean “surgical interventions”? Otherwise, the categories are not mutually exclusive.

We edited the term to explain “surgical interventions”.

24. Results: Characteristics: “77 received funding (67.5%)” this value is not completely correct, as 21 did not mention funding but may have been funded. Suggest to re-organize to: “Twenty-one studies did not include a funding statement (21/114, 18.4%). Of the remaining, 77 received funding (67.5%), with public funding …”

We corrected this statement to “Of the remaining, 77 received funding (67.5%), with public funding being the most common form of funding (44/114, 38.6%). Twenty-one studies did not include a funding statement (21/114, 18.4%), and 16 reported no funding (16/114, 14.04%)”.

25. Results: Table 2: Journal Impact Factor, M (SD): What is the value in the fourth column? If it is an odds ratio, what was the reference?

The fourth column was intended to be the odds ratio, interpreted in this situation as any increase in the impact factor, the odds of including spin being positive increase by factor 0.77 (or decrease by 0.23). The reference is 1.0.

26. Results: Table 3: In the right column, please indicate the items where partial yes was not applicable.

We provided a legend on table 3 in order to clarify which items where partial yes was not applicable.

27. Conclusion: Please change to “whose abstracts mostly portray the findings of the review’s full-text accurately.” to reflect the results adequately.

We edited the conclusion by stating “When comparing our results to other fields of medicine, most of the field of diabetic neuropathy research publishes systematic reviews whose abstracts portray the findings of the review’s full-text”.
