# Supplementary material for: The presence of spin in systematic reviews focused on diabetic neuropathy: A cross-sectional analysis
Source: PLoS One. 2022 Sep 26;17(9):e0274744. doi: 10.1371/journal.pone.0274744 (PMC9512415; doi:10.1371/journal.pone.0274744)
Supplement: Attachment — Submitted filename: Response to the Reviewers- The Second Rebuttal Letter.docx [file pone.0274744.s002.docx]

Response to reviewers

Journal Requirements:

PRISMA flow diagram

Original comment: Please provide enough information so a reader can reproduce your process of using automation tools that were used to screen records, and so readers can assess the reliability of doing this.

Author response: The PRISMA flow diagram is updated to where the reader will be able to reproduce the process of screening records.

New comment: There is still a vague reference to automation in the flow diagram, and a new detail in the methods that "96 because they were ineligible by automation tools". Which automation tools? How did they determine ineligibility? Why were they ineligible? How did you determine you could trust the algorithms?

During the exclusion criteria, Rayyan had an automation feature that enabled automatic exclusion criteria for any studies which didn’t pertain to diabetic neuropathy treatment. These criteria would be labeled; RAYYAN-LABELS. They are followed by specific exclusion criteria (ulcer, multiple diseases nonspecific to diabetes, literature review, etc.) which were determined at the beginning of the study. Any studies that met these specific exclusion criteria were rejected. For example, during the Rayyan screen if there was an article titled “diabetes complications for retinopathy” our Rayyan automation feature would have immediately rejected this article because it does not meet our inclusion criteria of diabetic neuropathy and instead meets the exclusion criteria of “retinopathy”. We have now added “^Automation Tools:” to our figure’s legend to clarify this.

Also, we have used Rayyan algorithms multiple times during our team's research projects, all of which maintained accurate reporting of data. The benefit of Rayyan is that it’s a semi-automation process which enables users to increase efficiency of screening abstracts and titles for systematic reviews, which was necessary due to the tedious nature of reviewing 1000 or more articles for eligibility. This document provides more information of why we used Rayyan for our systematic review analyses: https://systematicreviewsjournal.biomedcentral.com/articles/10.1186/s13643-016-0384-4

Thank you for providing access to your protocol, and for its comprehensiveness for reproducibility.

In the protocol, "To promote the reproducibility of our study, the study protocol, raw data, analysis scripts, data dictionaries, training videos, and extraction forms will be deposited on Open Science Framework." Do you plan to publish analysis scripts and data dictionaries in the repository?

At this time, any supplementary information formed for the paper was intended to be implemented in the protocol itself and uploaded to Open Science Frame to enable reproducibility. We will also provide our analysis script in the new submission. Our current analysis script is titled “Stata Code” and is uploaded to the OSF.

Thank you for attaching your data. It is not clear whether the editorial software converted the data files to PDF form, but it is extremely difficult to review these as PDFs. For instance, there are different colors in cells, it is not clear if there is a data dictionary describing what these colors mean, and I cannot read most of the text in the cells. Please include them as spreadsheets either as supplemental files or in your OSF repository.

This information will now be uploaded to the OSF repository to ensure transparency. A legend was also added to explain the variation of colors for each cell with Orange = Excluded, Red = Unable to find full length, English text.

The current data availability statement "All relevant data are within the manuscript" is not clear; if the data will be supplementary files or in the repository, this can be stated.

The supplementary files will now be uploaded to OSF.

Reviewer #2: The authors have satisfactorily addressed most of my previous comments. However, some important comments need further attention before their manuscript is accepted for publication.

1. Unfortunately I could not find the CREDIT Statement of authors' contributions. Furthermore, in the marked version of the manuscript, it appears to me that the order of authors has been changed since the initial submission. Please ensure that authorship is consistent in both manuscript versions (marked and unmarked). If the order of authors has indeed changed, this should also be reflected in the CREDIT statement (i.e. did they perform major work in the revision?).

The CREDIT statement has been updated and added to the manuscript. There was a reported discrepancy between the current credit author contribution statement and the previous author contribution statement. I have contacted the journal to find out what the specific discrepancy was for the contributions of each author. We can say definitively, however, that the current statement listed in the manuscript and the supplemental CREDIT Statement of authors’ contribution is the correct version. The previous version was inaccurate in listing each author's contribution and the current one is what we will use for this submission. Any additional major work performed in the revision is reflected in the current author statement.

2. Data sharing/transparency: The OSF page is now openly accessible, which is good. I was also pleased to see that you submitted the list of in- and excluded studies, details of included studies and spin ratings, and AMSTAR ratings as supplemental files. However, the current format (Excel sheets converted to PDF) is not fully readable. Without a legend it is also unclear what the colors mean. My understanding is that this is not the authors' fault, but the submission system (editorial manager) converts all document types to PDF. I therefore suggest that you convert the files to Word documents with legends and ensure every cell is fully readable before submitting them. Additionally (or instead) you may want to upload your Excel files to OSF.

The data files have been uploaded to OSF. Keys have been added to aid comprehension. We have placed all the tables in OSF so that they are legible.

3. In the abstract's conclusion there was a misunderstanding about my comment. I did not mean "to reflect the results adequately”. To be part of the sentence. However, I leave it up to the authors if they want to change it.

We have decided to leave the statement as is.

4. PRIMSA compliance as a subheading appears to be misleading. Suggest to move this section after "Spin data extraction" and name it "Data extraction of general characteristics" or similarly.

These corrections have been made. We moved the section after “Spin data extraction” and named it “Data Extraction of general characteristics”.

5. The odds ratio for JIF is still unclear to me; did you take the mean JIF and calculated the odds for journals below and above the mean JIF? If yes, please insert two rows (as for the other characteristics) and indicate which the reference row is.

We used JIF as a continuous variable. We provided the mean JIF among all publications included in our study in the first column of the table. We then used the continuous JIF variable to measure the association of JIF and inclusion of spin in the abstract of a manuscript. The interpretation is thus: For each 1 point increase in JIF, there is a 25% reduction in the odds that an abstract will contain spin (OR: 0.75 (0.50-1.12).

Please review your reference list to ensure that it is complete and correct. If you have cited papers that have been retracted, please include the rationale for doing so in the manuscript text, or remove these references and replace them with relevant current references. Any changes to the reference list should be mentioned in the rebuttal letter that accompanies your revised manuscript. If you need to cite a retracted article, indicate the article’s retracted status in the References list and also include a citation and full reference for the retraction notice.

To provide complete transparency for the updated references, we will be listing the original manuscripts list of references, with any changes or alterations to the current references being listed underneath the changed references. After this, we will provide the information for the added references to the current manuscript.

**References:**

1.	[National Diabetes Statistics Report, 2020. 28 Sep 2020 [cited 30 Jun 2021]. Available:](http://paperpile.com/b/I3oh8f/LmEpk) <https://www.cdc.gov/diabetes/data/statistics-report/index.html>

2.	[CDC. Diabetes and Nerve Damage. 14 May 2021 [cited 30 Jun 2021]. Available:](http://paperpile.com/b/I3oh8f/LkekC) <https://www.cdc.gov/diabetes/library/features/diabetes-nerve-damage.html>

3.	[Iqbal Z, Azmi S, Yadav R, Ferdousi M, Kumar M, Cuthbertson DJ, et al. Diabetic Peripheral Neuropathy: Epidemiology, Diagnosis, and Pharmacotherapy. Clin Ther. 2018;40: 828–849. doi:](http://paperpile.com/b/I3oh8f/I0phc)[10.1016/j.clinthera.2018.04.001](http://dx.doi.org/10.1016/j.clinthera.2018.04.001)

4.	[Murad MH, Asi N, Alsawas M, Alahdab F. New evidence pyramid. Evid Based Med. 2016;21: 125–127. doi:](http://paperpile.com/b/I3oh8f/588PB)[10.1136/ebmed-2016-110401](http://dx.doi.org/10.1136/ebmed-2016-110401)

5.	[Impellizzeri FM, Bizzini M. Systematic review and meta-analysis: a primer. Int J Sports Phys Ther. 2012;7: 493–503. Available:](http://paperpile.com/b/I3oh8f/pdYmz) <https://www.ncbi.nlm.nih.gov/pubmed/23091781>

6.	[Yavchitz A, Ravaud P, Altman DG, Moher D, Hrobjartsson A, Lasserson T, et al. A new classification of spin in systematic reviews and meta-analyses was developed and ranked according to the severity. J Clin Epidemiol. 2016;75: 56–65. doi:](http://paperpile.com/b/I3oh8f/PoSyE)[10.1016/j.jclinepi.2016.01.020](http://dx.doi.org/10.1016/j.jclinepi.2016.01.020)

7.	[Kinder NC, Weaver MD, Wayant C, Vassar M. Presence of “spin” in the abstracts and titles of anaesthesiology randomised controlled trials. Br J Anaesth. 2019;122: e13–e14. doi:](http://paperpile.com/b/I3oh8f/w133y)[10.1016/j.bja.2018.10.023](http://dx.doi.org/10.1016/j.bja.2018.10.023)

8.	[Reynolds-Vaughn V, Riddle J, Brown J, Schiesel M, Wayant C, Vassar M. Evaluation of Spin in the Abstracts of Emergency Medicine Randomized Controlled Trials. Ann Emerg Med. 2019. doi:](http://paperpile.com/b/I3oh8f/Xepnj)[10.1016/j.annemergmed.2019.03.011](http://dx.doi.org/10.1016/j.annemergmed.2019.03.011)

9.	[Marcelo A, Gavino A, Isip-Tan IT, Apostol-Nicodemus L, Mesa-Gaerlan FJ, Firaza PN, et al. A comparison of the accuracy of clinical decisions based on full-text articles and on journal abstracts alone: a study among residents in a tertiary care hospital. Evid Based Med. 2013;18: 48–53. doi:](http://paperpile.com/b/I3oh8f/nhWFp)[10.1136/eb-2012-100537](http://dx.doi.org/10.1136/eb-2012-100537)

These three references 7-9, were placed in the incorrect order. They are now correctly arranged in the current version of the manuscript in the order of Marcelo et al. 2013, Kinder et al. 2019, and Reynold et al. 2019.

10.	[Khan A, Riley H, Ottwell RL, Anderson M. Diabetic Neuropathy Spin. 2020 [cited 30 Jun 2021]. Available:](http://paperpile.com/b/I3oh8f/pLQKB) <https://osf.io/7e5nd/>

11.	[Beller EM, Glasziou PP, Altman DG, Hopewell S, Bastian H, Chalmers I, et al. PRISMA for Abstracts: reporting systematic reviews in journal and conference abstracts. PLoS Med. 2013;10: e1001419. doi:](http://paperpile.com/b/I3oh8f/me0Tz)[10.1371/journal.pmed.1001419](http://dx.doi.org/10.1371/journal.pmed.1001419)

12.	[Murad MH, Wang Z. Guidelines for reporting meta-epidemiological methodology research. Evid Based Med. 2017;22: 139–142. doi:](http://paperpile.com/b/I3oh8f/BK1f3)[10.1136/ebmed-2017-110713](http://dx.doi.org/10.1136/ebmed-2017-110713)

13.	[Rayyan QCRI. [cited 10 Sep 2019]. Available:](http://paperpile.com/b/I3oh8f/FiJFi) <https://rayyan.qcri.org/reviews/81224>

14.	[Moher D, Shamseer L, Clarke M, Ghersi D, Liberati A, Petticrew M, et al. Preferred reporting items for systematic review and meta-analysis protocols (PRISMA-P) 2015 statement. Syst Rev. 2015;4: 1. doi:](http://paperpile.com/b/I3oh8f/uQFcb)[10.1186/2046-4053-4-1](http://dx.doi.org/10.1186/2046-4053-4-1)

15.	[Introduction to Systematic Review and Meta-Analysis. [cited 30 Jun 2021]. Available:](http://paperpile.com/b/I3oh8f/zhSiB) <https://www.coursera.org/learn/systematic-review>

This reference was updated to its correct citation: Li T, Dickerson K. Introduction to systematic review and meta-analysis [Internet]. Coursera. John Hopkins University; [cited 2022Apr12]. Available from: https://www.coursera.org/learn/systematic-review

16.	[Lorenz RC, Matthias K, Pieper D, Wegewitz U, Morche J, Nocon M, et al. A psychometric study found AMSTAR 2 to be a valid and moderately reliable appraisal tool. J Clin Epidemiol. 2019;114: 133–140. doi:](http://paperpile.com/b/I3oh8f/Aaz3m)[10.1016/j.jclinepi.2019.05.028](http://dx.doi.org/10.1016/j.jclinepi.2019.05.028)

This reference is now replaced with: Shea BJ, Reeves BC, Wells G, Thuku M, Hamel C, Moran J, et al. Amstar 2: A critical appraisal tool for systematic reviews that include randomised or non-randomised studies of healthcare interventions, or both [Internet]. The BMJ. British Medical Journal Publishing Group; 2017 [cited 2021Jun21]. Available from: https://www.bmj.com/content/358/bmj.j4008

The reason for replacing the previous reference was to provide more information for our readers of AMSTAR-2. The previous reference provided excellent information for AMSTAR, but the newer reference was better suited toward our manuscript.

17.	[Ottwell R, Rogers TC, Michael Anderson J, Johnson A, Vassar M. Evaluation of Spin in the Abstracts of Systematic Reviews and Meta-Analyses Focused on the Treatment of Acne Vulgaris: Cross-Sectional Analysis. JMIR Dermatology. 2020;3: e16978. doi:](http://paperpile.com/b/I3oh8f/NYrB8)[10.2196/16978](http://dx.doi.org/10.2196/16978)

18.	[Liberati A, Altman DG, Tetzlaff J, Mulrow C, Gøtzsche PC, Ioannidis JPA, et al. The PRISMA statement for reporting systematic reviews and meta-analyses of studies that evaluate healthcare interventions: explanation and elaboration. BMJ. 2009;339. doi:](http://paperpile.com/b/I3oh8f/tjq8n)[10.1136/bmj.b2700](http://dx.doi.org/10.1136/bmj.b2700)

19.	[StataCorp. 2017. Stata Statistical Software: Release 15. College Station, TX: StataCorp LLC.](http://paperpile.com/b/I3oh8f/O0YFV)

20.	[Lunn MPT, Hughes RAC, Wiffen PJ. Duloxetine for treating painful neuropathy, chronic pain or fibromyalgia. Cochrane Database Syst Rev. 2014; CD007115. doi:](http://paperpile.com/b/I3oh8f/QMYjA)[10.1002/14651858.CD007115.pub3](http://dx.doi.org/10.1002/14651858.CD007115.pub3)

21.	[Fanelli D, Ioannidis JPA. US studies may overestimate effect sizes in softer research. Proc Natl Acad Sci U S A. 2013;110: 15031–15036. doi:](http://paperpile.com/b/I3oh8f/GnClv)[10.1073/pnas.1302997110](http://dx.doi.org/10.1073/pnas.1302997110)

22.	[Savovic J, Turner RM, Mawdsley D, Jones HE, Beynon R, Higgins JPT, et al. Association Between Risk-of-Bias Assessments and Results of Randomized Trials in Cochrane Reviews: The ROBES Meta-Epidemiologic Study. Am J Epidemiol. 2018;187: 1113–1122. doi:](http://paperpile.com/b/I3oh8f/jrJFk)[10.1093/aje/kwx344](http://dx.doi.org/10.1093/aje/kwx344)

23.	[Austin J, Smith C, Natarajan K, Som M, Wayant C, Vassar M. Evaluation of spin within abstracts in obesity randomized clinical trials: A cross-sectional review. Clin Obes. 2019;9: e12292. doi:](http://paperpile.com/b/I3oh8f/7yEYe)[10.1111/cob.12292](http://dx.doi.org/10.1111/cob.12292)

24.	[Jellison S, Roberts W, Bowers A, Combs T, Beaman J, Wayant C, et al. Evaluation of spin in abstracts of papers in psychiatry and psychology journals. BMJ Evid Based Med. 2019. doi:](http://paperpile.com/b/I3oh8f/3Wdwh)[10.1136/bmjebm-2019-111176](http://dx.doi.org/10.1136/bmjebm-2019-111176)

25.	[Wayant C, Margalski D, Vaughn K, Vassar M. Evaluation of spin in oncology clinical trials. Crit Rev Oncol Hematol. 2019;144: 102821. doi:](http://paperpile.com/b/I3oh8f/HytUV)[10.1016/j.critrevonc.2019.102821](http://dx.doi.org/10.1016/j.critrevonc.2019.102821)

26.	[Heigle B, Kee M, Ottwell R, Arthur W, Brame L, Wright DN, et al. Spin the Abstracts of Systematic Reviews and Meta-Analyses Regarding the Treatment of Ménière’s Disease. Ann Otol Rhinol Laryngol. 2021; 34894211000493. doi:](http://paperpile.com/b/I3oh8f/kp7xH)[10.1177/00034894211000493](http://dx.doi.org/10.1177/00034894211000493)

27.	[Demla S, Shinn E, Ottwell R, Arthur W, Khattab M, Hartwell M, et al. Evaluation of spin in the abstracts of systematic reviews and meta-analyses focused on cataract therapies. Am J Ophthalmol. 2021. doi:](http://paperpile.com/b/I3oh8f/2sAny)[10.1016/j.ajo.2021.03.032](http://dx.doi.org/10.1016/j.ajo.2021.03.032)

28.	[Reddy AK, Shepard S, Ottwell R, Thompson J, Price C, Arthur W, et al. Over 30% of systematic reviews and meta-analyses focused on rotator cuff tear treatments contained spin in the abstract. Arthroscopy. 2021. doi:](http://paperpile.com/b/I3oh8f/sKLmQ)[10.1016/j.arthro.2021.03.066](http://dx.doi.org/10.1016/j.arthro.2021.03.066)

29.	[Jones C, Rulon Z, Arthur W, Ottwell R, Checketts J, Detweiler B, et al. Evaluation of spin in the abstracts of systematic reviews and meta-analyses related to the treatment of proximal humerus fractures. J Shoulder Elbow Surg. 2021. doi:](http://paperpile.com/b/I3oh8f/9wT5y)[10.1016/j.jse.2020.11.026](http://dx.doi.org/10.1016/j.jse.2020.11.026)

30.	[Rucker B, Umbarger E, Ottwell R, Arthur W, Brame L, Woodson E, et al. Evaluation of Spin in the Abstracts of Systematic Reviews and Meta-Analyses Focused on Tinnitus. Otol Neurotol. 2021. doi:](http://paperpile.com/b/I3oh8f/GHbKC)[10.1097/MAO.0000000000003178](http://dx.doi.org/10.1097/MAO.0000000000003178)

31.	[Balcerak G, Shepard S, Ottwell R, Arthur W, Hartwell M, Beaman J, et al. Evaluation of spin in the abstracts of systematic reviews and meta-analyses of studies on opioid use disorder. Subst Abus. 2021; 1–9. doi:](http://paperpile.com/b/I3oh8f/0EC8J)[10.1080/08897077.2021.1904092](http://dx.doi.org/10.1080/08897077.2021.1904092)

32.	[Verble C, Cloeter M, Ottwell R, Arthur W, Hartwell M, Carr B, et al. Evaluation of spin in the abstracts of systematic reviews and meta-analyses focused on treatments of alcohol use disorder. Am J Drug Alcohol Abuse. 2021; 1–10. doi:](http://paperpile.com/b/I3oh8f/LXeNb)[10.1080/00952990.2021.1903022](http://dx.doi.org/10.1080/00952990.2021.1903022)

All of these references have been removed from the current manuscript. During the new revision, we reviewed our conclusion and felt a more concise version was in order. We felt as though we had already established studies reviewing over spin through various fields of medicine, and that it would be redundant to reference 10 additional studies which stated the same point.

33.	[Lazarus C, Haneef R, Ravaud P, Hopewell S, Altman DG, Boutron I. Peer reviewers identified spin in manuscripts of nonrandomized studies assessing therapeutic interventions, but their impact on spin in abstract conclusions was limited. J Clin Epidemiol. 2016;77: 44–51. doi:](http://paperpile.com/b/I3oh8f/BGSjn)[10.1016/j.jclinepi.2016.04.012](http://dx.doi.org/10.1016/j.jclinepi.2016.04.012)

34.	[Cumpston M, Li T, Page MJ, Chandler J, Welch VA, Higgins JP, et al. Updated guidance for trusted systematic reviews: a new edition of the Cochrane Handbook for Systematic Reviews of Interventions. Cochrane Database Syst Rev. 2019;10: ED000142. doi:](http://paperpile.com/b/I3oh8f/J906c)[10.1002/14651858.ED000142](http://dx.doi.org/10.1002/14651858.ED000142)

We added these two references to show evidence of spin in other fields of medicine in relation to ours. We wanted to establish this in our introduction to provide more context for the purpose of our study.

10.	Cooper CM, Gray HM, Ross AE, Hamilton TA, Bea Downs J, Wayant C, Vassar M; Evaluation of spin in the abstracts of otolaryngology randomized controlled trials [Internet]. The Laryngoscope. U.S. National Library of Medicine; 2018 [cited 2022Mar7]. Available from: https://pubmed.ncbi.nlm.nih.gov/30578543/

11.	Roberts WB,Cooper CM,Khattab M;Neff P;Wildes D,Wayant C, Vassar M; Evaluation of "spin" in the abstracts of randomized controlled trial reports in Cardiology [Internet]. The Journal of the American Osteopathic Association. U.S. National Library of Medicine; 2020 [cited 2021May24]. Available from: https://pubmed.ncbi.nlm.nih.gov/32961554/
